# Caffeine treatment started before injury reduces hypoxic–ischemic white-matter damage in neonatal rats by regulating phenotypic microglia polarization

**DOI:** 10.1038/s41390-021-01924-6

**Published:** 2022-02-26

**Authors:** Liu Yang, Xuefei Yu, Yajun Zhang, Na Liu, Xindong Xue, Jianhua Fu

**Affiliations:** 1 Department of Pediatrics, Shengjing Hospital of China Medical University, 110004 Shenyang, Liaoning P.R. China; 2 Department of Pediatrics, The Second Hospital of Dalian Medical University, 116021 Dalian, Liaoning P.R. China; 3 Department of Anesthesiology, Dalian Municipal Maternal and Child Health Care Hospital, 116021 Dalian, Liaoning P.R. China

## Abstract

**Background:**

Reducing neuroinflammatory damage is an effective strategy for treating white-matter damage (WMD) in premature infants. Caffeine can ameliorate hypoxia–ischemia-induced brain WMD; however, its neuroprotective effect and mechanism against hypoxic–ischemic WMD remain unclear.

**Methods:**

We used 3-day-old Sprague–Dawley rats to establish a model of cerebral hypoxia–ischemia-induced brain WMD after unilateral common carotid artery ligation and hypoxia exposure (8% O_2_ + 92% N_2_) for 2.5 h. Mechanism experiments were conducted to detect M1/M2 polarization and activation of microglia and NLRP3 inflammasome.

**Results:**

Caffeine inhibited NLRP3 inflammasome activation, reduced microglial Iba-1 activation, inhibited microglia M1 polarization, and promoted microglia M2 polarization by downregulating CD86 and iNOS protein expression, inhibiting the transcription of the proinflammatory TNF-α and IL-1β, upregulating CD206 and Arg-1 expression, and promoting the transcription of the anti-inflammatory factors IL-10 and TGF-β. Importantly, we found that these caffeine-mediated effects could be reversed after inhibiting A2aR activity.

**Conclusions:**

Caffeine improved long-term cognitive function in neonatal rats with hypoxic–ischemic WMD via A2aR-mediated inhibition of NLRP3 inflammasome activation, reduction of microglial activation, regulation of the phenotypic polarization of microglia and the release of inflammatory factors, and improvement of myelination development.

**Impact:**

- The direct protective effect of caffeine on hypoxic–ischemic white-matter damage (WMD) and its mechanism remains unclear. This study elucidated this mechanism using neonatal rats as an animal model of hypoxia–ischemia-induced cerebral WMD.
- The findings demonstrated caffeine as a promising therapeutic tool against immature WMD to protect neonatal cognitive function.
- We found that caffeine pretreatment reduced WMD in immature brains via regulation of microglial activation and polarization by adenosine A2a receptor, thereby, providing a scientific basis for future clinical application of caffeine.

## Introduction

It is estimated that 9 million (60%) of the 15 million premature babies born annually will suffer life-long physical or neurological disabilities.^1–3^ Compared with full-term infants, premature infants are more susceptible to unfavorable perinatal environments that cause organ damage, especially brain damage, due to their small gestational age and the relatively immature development of various organs.^4^ White-matter damage (WMD) is the most common type of brain injury in premature infants and presents pathological features that include decreased myelin sheath, oligodendrocyte-maturation disorder, synaptic dysplasia, and neuroinflammation, which ultimately lead to cerebral palsy, cognition, and neuroinflammation, as well as barriers in language and behavioral capabilities.^5,6^ Among these features, inflammation plays a central role in the development of brain injury in newborns,^7^ and neonatal hypoxia–ischemia can trigger an inflammatory response.^8^ Previous studies support the hypothesis that hypoxia–ischemia-induced systemic inflammation impairs oligodendrocyte maturation through neuroinflammatory processes, including microglial activation.^9,10^ At a mechanistic level, activated microglia may damage white matter after hypoxic–ischemic damage via sustained elevation of proinflammatory molecules, such as tumor necrosis factor (TNF)-α, interleukin (IL)-1β, IL-6, and complement pathways.^11,12^ Therefore, reducing neuroinflammatory damage is considered an effective strategy for the treatment of neonatal WMD.

Microglia are key players in neuroinflammation, which is positively regulated by activation of nucleotide-binding oligomerization domain-like receptor (NLR) family members, such as NLR- and pyrin domain-containing 3 (NLRP3).^13,14^ Microglia can be activated through different signaling pathways and polarized into proinflammatory (M1) or anti-inflammatory (M2) phenotypes, from which proinflammatory or anti-inflammatory mediators are released to either aggravate or promote brain damage or repair.^15–17^ Previous studies reported increased microglial activation and differentiation to an M1 phenotype in neonatal hypoxic–ischemic brain injury, whereas inhibiting inflammation-related microglial activation or changing to an M2 phenotype reduced WMD and improved cognitive function.^18,19^ Therefore, regulating microglial polarization represents a potential strategy for treating WMD-related diseases.

Caffeine is a methylxanthine drug that has been used in the neonatal intensive care unit to treat neonatal apnea for >30 years.^20,21^ Caffeine exerts anti-oxidative stress, anti-inflammation, and anti-apoptosis activities, as well as free radical-scavenging capabilities.^22–26^ Studies indicate that caffeine can resist hypoxia–ischemia-induced brain WMD by reducing the apoptosis of developing brain neurons,^27–29^ reducing myelination disorders.^30^ Additionally, reports indicate a key role for adenosine A2a receptor (A2aR) in neuroinflammation.^31^ Colella et al.^32^ revealed that the A2aR agonist CGS-21680 promotes increased levels of CD73 protein and the proinflammatory cytokines IL-1β, IL-6, inducible nitric oxide synthase (iNOS), and TNF-α in brain neuroinflammation rat models. As an A2aR antagonist, caffeine prevents these activities and neuroinflammation^33^; however, the direct protective effect of caffeine against hypoxic–ischemic WMD and its mechanism in neonatal rats remain unclear. Moreover, whether caffeine can antagonize A2aR to prevent neuroinflammation and regulate microglial activation and phenotypic polarization remains to be elucidated.

In this study, we evaluated the anti-neuro-inflammatory effects of caffeine as a possible neuroprotective strategy against hypoxic–ischemic WMD in premature infants using neonatal rats as an animal model.

## Materials and methods

### Animals and ethics statement

All animal experiments were approved by the Animal Ethical Committee of China Medical University (Shenyang, China; 2017PS140K). Perinatal Sprague–Dawley (SD) rats were purchased from Liaoning Changsheng Biotechnology Co., Ltd (Liaoning, China) and housed in facility with a 12-h light/dark cycle and with free access to food and water.

### Establishment of the hypoxia–ischemia-induced brain WMD model

Animal models of neonatal WMD were established according to previously described methods.^34,35^ Briefly, 3-day-old SD rats (male and female) were anesthetized by isoflurane inhalation and fixed on the operating table in the supine position. The left common carotid artery was then exposed under a dissecting microscope, and the hypoxia–ischemia (HI) group was permanently ligated with 2.0 sterile-needle sutures. At both ends of the artery, the blood vessel was cut in the middle of the two ligature points, and the wound was sutured. The operation time was 8 min to 10 min. After the operation, the rat was awake and sent back to the mother to recover for 1 h, followed by placement in a hypoxic box (kept in a water bath at a constant temperature of 37 °C). Mixed gas (8% O_2_ + 92% N_2_) was continuously input into the box for 2.5 h, with a gas flow of 2 L/min and an oxygen concentration maintained at 8%. Rats in the sham operation group had the left common carotid artery separated without ligation and hypoxia treatment. The body temperature of rats in all procedures was maintained between 36 °C and 37 °C.

The total number of newborn rats used in the experiment was between 10 and 16 in each litter, and the birth weight was ~7.3 g (7.26 ± 0.325). All the rats get cross-fostered between dams. A total of 420 pups were used for this study. The animals were randomly divided into four groups (*n* = 10/group): sham, model (HI), caffeine treatment (caffeine), and caffeine+CGS21680 treatment (caffeine + CGS21680). From days 2 to 6 after birth, regular intraperitoneal injection of 20 mg/kg/day caffeine citrate or an equal volume of normal saline was injected for 5 consecutive days. The caffeine+CGS21680 group received intraperitoneal injection of 2 mg/kg/day CGS21680 along with 20 mg/kg/day caffeine citrate. The caffeine citrate used in this study was produced by Casey Pharmaceuticals (Parma, Italy), and CGS21680 was produced by Sigma-Aldrich (C141; Lyon, France). The body weight of the rats was recorded on days 0, 3, 7, 14, 21, and 28 after the establishment of the rat model. The specimens were collected 7 (P10), 14 (P17), and 21 days (P24) after model establishment (specimens from the caffeine + CGS21680 group were only collected on P17), and six rats from each group were randomly selected at each time point for inclusion in statistical analysis of histology from paraffin sections, western blot results, polymerase chain reaction (PCR) results, and enzyme-linked immunosorbent assay (ELISA) results. Additionally, all 10 rats in each group underwent the Morris water maze (MWM) test after model establishment, with the results from six of the rats used for statistical analysis.

### MWM test

**Fig. 1:**
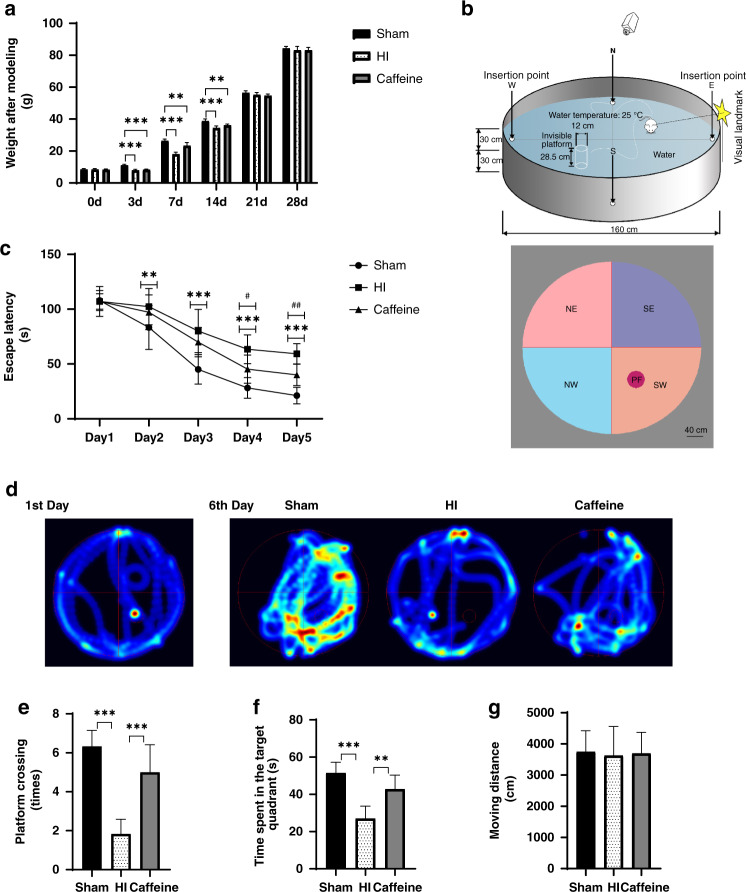
Caffeine reduces cognitive impairment caused by hypoxic–ischemic WMD in neonatal rats without influencing body weight. **a** Comparison of body weights in each group on days 3, 7, 14, 21, and 28 after model establishment and prior to the experiment. **b** Schematic diagram of the water maze experimental device. NE northeast, SE southeast, NW northwest, SW southwest, PF platform. **c** The escape latencies of rats in the training trials for the hidden platform task. **d** Heat graph of the representative pathways on the first and last training days for the hidden platform task for each group. **e** Frequency of platform crossing (times) in the probe trial. **f** Time spent in the target quadrant during the probe trial. **g** Moving distance (cm) in the probe trial. Scale bar = 40 cm. Data represent the mean ± SEM. Statistical analyses included two-way and one-way ANOVA, followed by Tukey’s test. *^/#^*P* < 0.05, **^/##^*P* < 0.01, ***P < 0.001. **a** #: HI vs. Caffeine group; *: Sham vs. HI group. Sham group (*n* = 6); HI group (*n* = 6); and Caffeine group (*n* = 6).

MWM tests were performed from days 28 to 33 after model establishment.^36^ The test comprised the following: a circular pool (diameter: 160 cm; height: 60 cm), a black inner wall, a movable platform (diameter: 12 cm; height: 28.5 cm), a computer, a camera, and an image and data acquisition and processing systems. Different graphics were used to mark the pool wall at the midpoints of the four quadrants. Before starting the experiment, water was poured into the pool to a depth of 30 cm, and the height of the platform was set to 1.5 cm below the water surface (Fig. 1b). The water temperature was maintained at 25 °C during testing. The test included two phases: acquisition training and probe trial. During training, the rats were trained continuously for 5 days (four times daily) to find a platform within 120 s. Once a rat found and stayed on the platform for 5 s, the training session was terminated. The time from entering the water to finding the platform represented the escape latency, and the system software automatically analyzed the swimming distance of the rat during this period. If the rat did not find the platform within 120 s, it was guided to rest on the platform for 20 s, and the escape latency was recorded as 120 s. On day 6, the platform was removed, and the probe trial was conducted. The rat was placed in the opposite quadrant and allowed to swim for 120 s, with data recorded by a video tracking system (Shanghai Mobile Datum Ltd., Shanghai, China). All tests were performed by researchers who were blinded to the experimental group. To evaluate the role of caffeine in cognitive impairment, the parameters of the MWM test, including escape latency, time spent in the target quadrant(s), frequency of platform crossing (times), and moving distance (cm), were evaluated.

### Hematoxylin and eosin (H&E) staining and measurement of ventricular area

At days 7, 14, and 21 after model establishment, the rats were anesthetized by isoflurane inhalation and perfused transcardially with 0.9% saline in distilled water, followed by perfusion with 4% paraformaldehyde in 0.1 M phosphate-buffered saline (PBS) and post-fixation of their brains in 4% formaldehyde in 0.01 M PBS for >48 h. The brains were then dehydrated, embedded in paraffin, and sliced to a thickness of 3 µm. The sections were then dewaxed with xylene, hydrated with ethanol, stained with hematoxylin (Solarbio, Beijing, China) for 10 min, rinsed with tap water for 30 min, and placed in eosin (Solarbio) for 3 min. After routine dehydration, transparentization, and sealing, the morphology of the ventricular region was observed at ×40 magnification using an optical microscope (Olympus, Tokyo, Japan), and the ventricular area was determined, as described previously.^27^ Digitized images were obtained that included the region of the lateral ventricle. The border of each ventricle for each section was outlined, and the cross-sectional ventricular areas were determined using Image-Pro software (Media Cybernetics, Bethesda, MD). The ventricular area was presented as the mean ± standard error of the mean (SEM) calculated from serial sections spanning the midstriatum.

### Immunohistochemistry (IHC) analysis

After routine slicing of brain tissue, baked slices were dewaxed with xylene and then hydrated with a gradient ethanol solution, followed by heating for 30 min in citrate buffer (pH 6.0) to repair the high-temperature antigen. Sections were treated with 3% hydrogen peroxide for 20 min and blocked with goat serum for 30 min, followed by incubation at 4 °C overnight with rabbit anti-myelin basic protein (MBP; monoclonal; 1:5000; ab218011; Abcam, Cambridge, UK), rabbit anti-allograft inflammatory factor 1 (AIF1)/ionized calcium-binding adaptor molecule 1 (Iba-1; polyclonal; 1:100; Cat.# DF6442; Affinity, Jiangsu, China), and rabbit anti-A2aR (polyclonal; 1:200; 51092-1-AP; Proteintech, Rosemont, IL). After rewarming and incubation with the appropriate streptavidin–horseradish peroxidase (HRP)-conjugated secondary antibody for 20 min at 37 °C, the samples were stained 3,3′-diaminobenzidine, re-dyed, dehydrated, transparentized, and sealed. Images were visualized and obtained using a light microscope (Olympus) and analyzed using the ImageJ software (NIH, Bethesda, MD). A global threshold was manually established for the signal, followed by quantification of the positive signal detected above the selected threshold for the determination of an average optical density value.

### Immunofluorescence

After deparaffinization and heat-mediated antigen retrieval, tissue sections were blocked with goat serum for 30 min at 37 °C and then incubated at 4 °C overnight with rabbit anti-CD86 (polyclonal; 1:200; 13395-1-AP; Proteintech, Rosemont, IL), rabbit anti-CD206 (polyclonal; 1:500; ab125028; Abcam), mouse anti-Iba-1 (1:100; ab15690; Abcam), or rabbit anti-NLRP3 (polyclonal; 1:200; ab214185; Abcam). After rewarming, sections were incubated with an Alexa Fluor 488-conjugated (1:200; ab150105; Abcam) or Alexa Fluor 594-conjugated (1:200; ab150076; Abcam) secondary antibodies for 4 h at 24–26 °C. All sections were then counterstained with 4′,6-diamidino-2-phenylindole, and images were visualized and obtained using a confocal laser-scanning microscope (C1; Nikon, Tokyo, Japan). Images were randomly selected, and NLRP3 levels were analyzed using the ImageJ software (NIH) to determine the average fluorescence intensity.

### Western blot

On days 7, 14, and 21 after HI injury, rats were euthanized and their brains harvested. After stripping the cortex, brain tissues were isolated from the ligation side paraventricular areas on the ice and stored at −80 °C. Samples were processed for western blot analysis, as described previously,^37^ and the membrane was incubated with rabbit anti-MBP (1:1000, Abcam), rabbit anti-AIF1/Iba-1 (1:1000; Affinity), rabbit anti-CD86 (1:1000; Proteintech), rabbit anti-CD206 (1:1000; Abcam), rabbit anti-iNOS (polyclonal; 1:1000; 18985-1-AP; Proteintech), rabbit anti-Arginase-1 (polyclonal; 1:5000; 16001-1-AP; Proteintech), rabbit anti-NLRP3 (1:1000; Abcam), rabbit anti-caspase-1 (monoclonal; 1:1000; ab207802; Abcam), rabbit anti-IL-1β (polyclonal; 1:1000; Cat.#:AF5103; Affinity), and rabbit anti-A2aR (1:500; Proteintech), with rabbit anti-β-tubulin (polyclonal; 1:5000; 10068-1-AP; Proteintech) used as a loading control. After incubation with HRP-conjugated goat anti-rabbit secondary antibody (1:5000; SA00001-2; Proteintech) and development using enhanced chemiluminescence reagents (Thermo Fisher Scientific, Waltham, MA), the intensities of all the bands were analyzed using ImageJ software (NIH) and normalized against β-tubulin.

### Real-time PCR

The expression of *Il1b*, *Tnfa*, *il10*, and *transforming growth factor-β* (*Tgfb*) mRNA in the ligation side paraventricular brain tissue was evaluated by reverse transcription (RT)-PCR. RNA samples were isolated from the left midbrain region of paraventricular brain tissue, which was then homogenized after grinding and centrifuged at 4 °C (14,000 rpm, 15 min). Total RNA was extracted using a TRIzol kit (Takara Bio, Dalian, China) and transcribed into cDNA, using HiScript QRT SuperMix for qPCR (+gDNA wiper; R123–01; Vazyme Biotech Co., Ltd., Nanjing, China). RT-PCR was performed using ChamQ universal SYBR qPCR master mix (Q711; Vazyme Biotech Co., Ltd.), and relative expression levels were determined using the 2^−ΔΔCT^ method, with *glyceraldehyde 3-phosphate dehydrogenase* (*Gapdh*) used as a reference. Primers were generated by Shanghai Biotechnology Service Co. (Shanghai, China) with the following sequences: *Il1b* forward 5′-ATCTCACAGCATCTCGACAAG-3′ and reverse 5′-CACACTAGCAGGTCGTCATCC-3′; *Tnfa* forward, 5′-GCATGATCCGAGATGTGGAACTGG-3′ and reverse 5′-CGCCACGAGCAGGAATGAGAAG-3′; *Il10* forward, 5′-CAAGGCAGTGGAGCAGGTGA-3′ and reverse, 5′-CCGGGTGGTTCAATTTTTCATT-3′; *Tgfb* forward 5′-GGCACCATCCATGACATGAAC-3′ and reverse 5′-GCCGTACACAGCAGTTCTCTG-3′; and *Gapdh* forward:5′-GACATGCCGCCTGGAGAAAC-3′ and reverse 5′-AGCCCAGGATGCCCTTTAGT-3′.

### Enzyme-linked immunosorbent assay

Determination of inflammatory factors in the ligation side paraventricular brain tissue was performed using ELISA. PBS was added to the brain tissue, which was then homogenized and centrifuged for 15 min at 3500 r.p.m. to obtain the supernatant. Levels of IL-1β (EK0393; Boster, Wuhan, China), TNF-α (EK0526; Boster), IL-10 (EK0418; Boster), and TGF-β (EK0514; Boster) were then detected by ELISA by measuring optical density at 450 nm using a microplate reader (Multiskan FC; Thermo Fisher Scientific).

### Imaging and analysis

The immune-stained sections were observed under Olympus (Melville, NY) BX51TF microscope, and images were taken from paraventricular white matter ranging from 1.5 mm before bregma to 0.5 mm after bregma. The detection areas were mainly corpus callosum (CC) and subventricular zone (SVZ) areas. For each group, we traced *n* = 6 cells of microglia (3 microglia were randomly from each rat for each group for each area) from each of the two previously quantified brain areas for each group. We performed Sholl analysis for the morphological evaluation of microglial cells. The immunoreactivity of microglial cells was quantified with the help of ImageJ software.

### Statistical analysis

Data are presented as the mean ± SEM. For normality assessment, Kolmogorov–Smirnov test with Dallal–Wilkinson–Lillie correction for *P* values was used. Data related to latency escape or time were analyzed using two-way repeated analysis of variance (ANOVA), with parametric one-way ANOVA with Tukey’s post-test used for comparisons of all other results. GraphPad Prism software (v.8.01; GraphPad Software, La Jolla, CA) was used for the analyses, and a *P* < 0.05 was considered statistically significant.

## Results

### Caffeine attenuates cognitive impairment related to hypoxic–ischemic WMD in neonatal rats without influencing body weight

We observed that the weights of neonatal rats with hypoxic–ischemic WMD decreased. On day 3 post-operation, the body weight of rats in the HI and caffeine groups decreased significantly as compared with that of rats in the sham group (*P* < 0.001). The weight difference between the sham, HI, and caffeine groups subsequently decreased in the following days (Fig. 1a) until no significant difference was observed on day 21 between the three groups.

In the MWM test, the escape latency of the HI group was significantly longer than that of the sham group at each time point (*P* < 0.001), with these times decreased by caffeine administration on days 4 and 5 (*P* < 0.05, *P* < 0.01, respectively) (Fig. 1c). In spatial probe trials, rats in the HI group showed a shorter exercise time in the target quadrant and exhibited a significantly reduced number of platform crossings (all *P* < 0.001), with each of these activities improved following caffeine administration (*P* < 0.01, *P* < 0.001, respectively) (Fig. 1d–f). Additionally, moving-distance results to assess athletic ability showed that the observed differences between groups were not caused by differences in athletic ability (Fig. 1g). These results indicated that caffeine reduced the cognitive dysfunction of neonatal rats with hypoxia–ischemia-induced WMD.

### Caffeine protects against hypoxic–ischemic WMD in neonatal rats

**Fig. 2:**
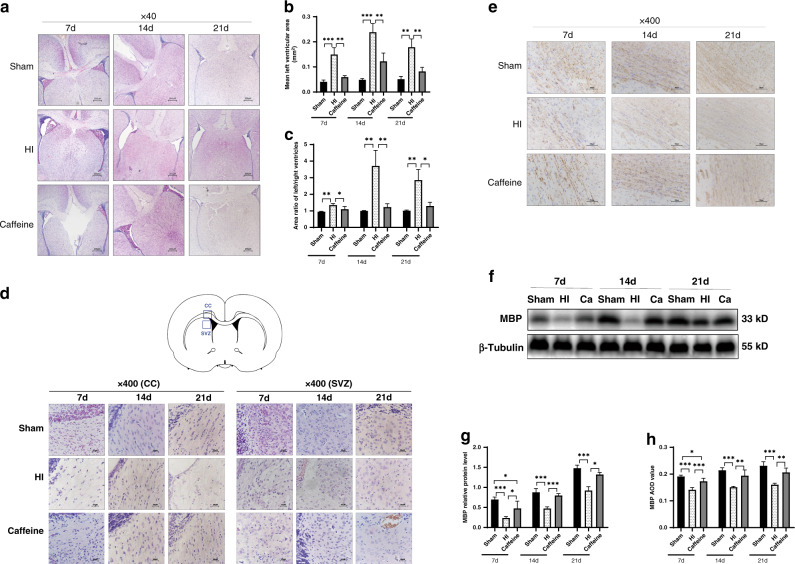
Protective effect of caffeine on hypoxic–ischemic WMD in neonatal rats. **a** Representative coronal views of the bilateral ventricles. Scale bar = 200 μm. **b** Mean ligated lateral (left) ventricular areas (mm^2^). **c** Area ratio of left/right ventricles. **d** H&E staining in the left corpus callosum (CC) area and subventricular zone (SVZ) at days 7, 14, and 21 after hypoxia–ischemia (HI) injury. Scale bar = 20 μm. **e** IHC staining showing MBP in the left corpus callosum at days 7, 14, and 21 after HI injury. Scale bar = 50 μm. **f** Western blot detection of MBP. **g** Analysis of relative MBP levels, with β-tubulin used for normalization. **h** AOD value of MBP. Data represent the mean ± SEM. Statistical analyses involved two-way ANOVA, followed by Tukey’s test. *P < 0.05, **P < 0.01, ***P < 0.001. Sham group (*n* = 6); HI group (*n* = 6); and Caffeine group (*n* = 6). Ca Caffeine.

We then observed the effect of caffeine on the expansion and development of cerebral ventricles in neonatal rat models of hypoxic–ischemic WMD by H&E staining. On days 7, 14, and 21 after model establishment, ligated lateral ventricles were enlarged in the HI group (Fig. 2a), whereas caffeine treatment improved the degree of ventricle enlargement (Fig. 2a). To assess the ventricle size, we analyzed and calculated the area of the left and right ventricles of three sets of continuous coronal views at the same level. Compared with that observed in the sham group, the area of the ligated lateral (left) ventricle was significantly increased in the HI group (*P* < 0.001, *P* < 0.01, respectively), and we observed clear changes in the asymmetry ratio of the left and right ventricles (all *P* < 0.01) on days 7, 14, and 21 after model establishment (Fig. 2b, c). Notably, these symptoms showed improvement following caffeine treatment (*P* < 0.05, *P* < 0.01, respectively; Fig. 2b, c). Compared with the sham group, at 7, 14, and 21 days after WMD, the white matter in the CC area and the SVZ of the HI group was lightly stained, the number of cells was reduced, and the structure was sparse, presenting a mesh-like change. This situation was improved after the application of caffeine. The increase in the number of cells in the paraventricular area and the orderly arrangement of the cells confirmed the protective effect of caffeine on white matter (Fig. 2d).

We then performed IHC and western blot analyses to determine the MBP content in the CC area of the ligated lateral hemisphere on days 7, 14, and 21 days after model establishment. IHC results showed that compared with the sham group, the density of MBP myelinating protein in the HI group was significantly reduced (all *P* < 0.001), with the density subsequently increased after caffeine administration (*P* < 0.01, *P* < 0.001, respectively; Fig. 2e, h). Western blot results showed that MBP levels in the HI group were significantly reduced relative to those in the sham group (all *P* < 0.001), and that caffeine treatment subsequently upregulated these levels (*P* < 0.05, *P* < 0.001, respectively; Fig. 2f, g). These data indicated that caffeine improved myelination development after hypoxia–ischemia.

### Caffeine inhibits activation of the NLRP3 inflammasome after hypoxic-ischemic WMD in neonatal rats

Under pathological conditions in the central nervous system (CNS), the NLRP3 inflammasome plays an important role in regulating inflammatory responses.^38^ To evaluate the inhibitory activity of caffeine against neuroinflammation in neonatal rats with WMD, we used immunofluorescence staining and western blot to detect NLRP3 levels and the levels of related proteins, including caspase-1 and IL-1β.

**Fig. 3:**
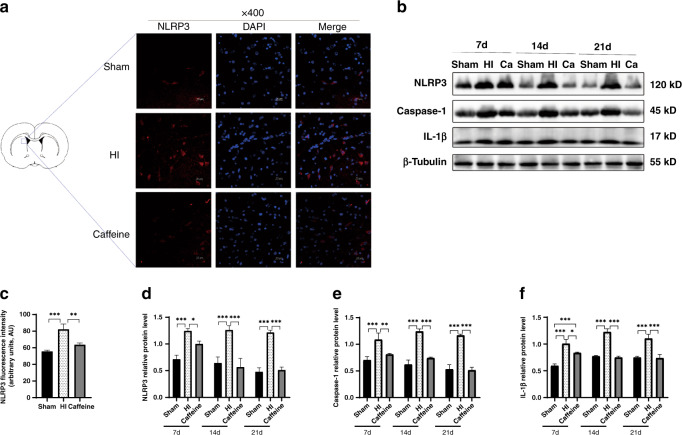
Caffeine inhibits activation of the NLRP3 inflammasome in neonatal rats with hypoxic–ischemic WMD. **a** Immunofluorescence staining and **c** fluorescence intensity analysis of NLRP3. Scale bar = 50 μm. **b** Western blot detection of NLRP3, caspase-1, and IL-1β. Analyses of relative **d** NLRP3, **e** caspase-1, and **f** IL-1β levels, with β-tubulin used for normalization. Data represent the mean ± SEM. Statistical analyses involved two-way and one-way ANOVA, followed by Tukey’s test. *P < 0.05, **P < 0.01, ***P < 0.001. Sham group (*n* = 6); HI group (*n* = 6); and Caffeine group (*n* = 6). Ca Caffeine.

Immunofluorescence results showed that 14 days after WMD, NLRP3 expression in the HI group was elevated relative to that in the sham group (*P* < 0.001), and that caffeine treatment partially inhibited this effect and reduced NLRP3 level (*P* < 0.01) (Fig. 3a, c). Additionally, western blot results showed that levels of NLRP3, caspase-1, and IL-1β were elevated to varying degrees on days 7, 14, and 21 days after WMD relative to those in the sham group (*P* < 0.001), but that caffeine treatment reduced these levels to varying degrees (*P* < 0.05, *P* < 0.01 or *P* < 0.001, respectively; Fig. 3b, d–f). These results suggested that caffeine reduced inflammasome activation via upregulation of NLRP3 expression and played an important role in inhibiting neuroinflammation.

### Caffeine reduces the number and M1/M2-polarization state of microglia after hypoxic–ischemic WMD in neonatal rats

Numerous studies show that pathogen infection of the CNS activates the formation of the NLRP3 inflammasome in microglia as a key sign of neuroinflammation.^39–41^ Therefore, we assessed the effect of caffeine on microglia following the establishment of the WMD model in neonatal rats.

**Fig. 4:**
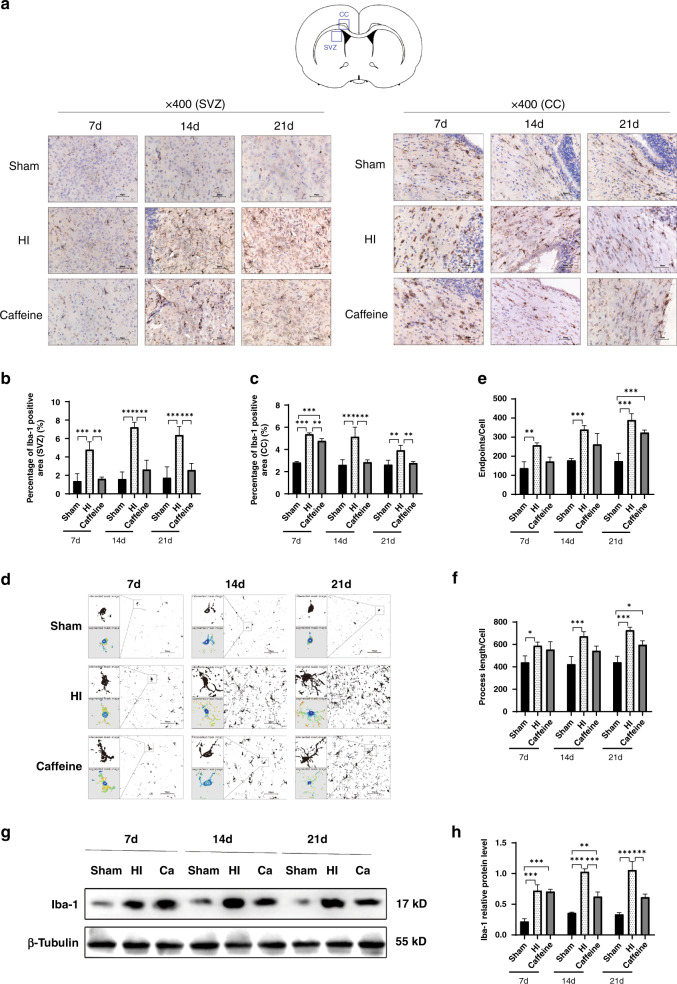
Caffeine reduces the number of activated microglia in neonatal rats with hypoxic–ischemic WMD. **a** IHC staining for Iba-1 in the CC and SVZ. Scale bar = 50 μm. **b** The percentage of Iba-1-positive area in the SVZ. **c** The percentage of Iba-1-positive area in the CC. **d** Morphological reconstructions of microglia (intersection and segmented mask). **e** Endpoints of microglia cells. **f** Process length of microglia cells. **g** Western blot detection of Iba-1. **h** Analysis of relative Iba-1 level, with β-tubulin used for normalization. Data represent the mean ± SEM. Statistical analyses involved two-way ANOVA, followed by Tukey’s test. *P < 0.05, **P < 0.01, ***P < 0.001. Sham group (*n* = 6); HI group (*n* = 6); Caffeine group (*n* = 6). Ca Caffeine.

We performed IHC and western blot analyses of Iba-1 in the ligated cerebral hemispheres of rats at 7, 14, and 21 days after WMD establishment to explore the role of caffeine in microglial activation. IHC results showed that compared with those in the sham group, we observed an increase in the number of microglia in the CC area and SVZ of the left cerebral hemispheres in the HI group (all *P* < 0.001); however, caffeine treatment partially inhibited this effect and reduced the area of microglia at each time point (*P* < 0.01, *P* < 0.001, respectively) (Fig. 4a–c). Then we analyzed the morphology of microglia. At 7, 14, and 21 days after WMD, microglia in the HI group formed amoeba morphology, and the number of endpoints and process length of branches per cell were increased in different degrees (*P* < 0.5, *P* < 0.05 or *P* < 0.01, respectively), but the above morphology did not improve significantly after caffeine treatment (Fig. 4d–f). Additionally, western blot results showed that Iba-1 levels at 7, 14, and 21 days after WMD increased significantly (all *P* < 0.001), but that caffeine administration significantly inhibited these increased levels on days 14 and 21 (all *P* < 0.001; Fig. 4g, h). These findings suggested that caffeine treatment downregulated microglial number and protein expression, but has less effect on the morphology of microglia.

**Fig. 5:**
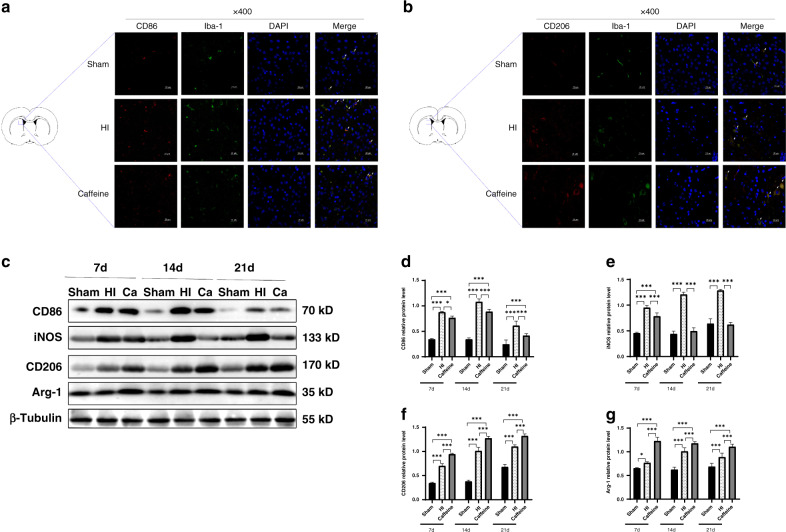
Caffeine regulates the M1/M2 polarization of microglia in neonatal rats with hypoxic–ischemic WMD. **a** Representative immunofluorescence image showing co-localization of CD86 (red) and Iba-1 (green) in the SVZ. Scale bar = 50 μm. **b** Representative immunofluorescence images showing co-localization of CD206 (red) and Iba-1 (green) in the SVZ. Scale bar = 50 μm. **c** Western blot detection of CD86, CD206, iNOS, and Arg-1. Analyses of relative **d** CD86, **e** iNOS, **f** CD206, and **g** Arg-1 levels, with β-tubulin used for normalization. Data represent the mean ± SEM. Statistical analyses involved two-way ANOVA, followed by Tukey’s test. *P < 0.05, **P < 0.01, ***P < 0.001. Sham group (*n* = 6); HI group (*n* = 6); Caffeine group (*n* = 6). Ca Caffeine.

Microglia assume either an M1 (proinflammatory) or M2 (anti-inflammatory) phenotype according to environmental cues. To determine the effect of caffeine on microglial polarization, we performed western blot analysis to detect changes in levels of M1-related markers (CD86, iNOS) and M2-related markers (CD206, Arg-1) at 7, 14, and 21 days after WMD establishment in neonatal rats. The results showed upregulated levels of both M1 and M2 markers in the HI group (all *P* < 0.001, except Arg-1 *P* < 0.05); however, caffeine treatment downregulated levels of M1 markers (*P* < 0.001, *P* < 0.05, respectively; Fig. 5c–e), while significantly upregulating those of M2 markers (all *P* < 0.001; Fig. 5c, f–g) at all time points.

We then used immunofluorescence to determine co-localization of polarized microglia according to M1 (CD86/Iba-1) and M2 (CD206/Iba-1) markers at 14 days after WMD establishment. Consistent with the western blot results, the number of M1-type microglia double-labeled with CD86 and Iba-1 in the SVZ was upregulated in the HI group relative to the sham group, with these numbers decreasing following caffeine treatment (Fig. 5a). By contrast, elevated numbers of M2-type microglia double-labeled with CD206 and Iba1 in the SVZ of the HI group were subsequently significantly upregulated further following caffeine treatment (Fig. 5b). These data indicated that caffeine inhibited microglial transformation to the M1 phenotype and promoted the acquisition of the M2 phenotype.

### Caffeine alters cytokine transcription and release in hypoxic–ischemic WMD neonatal rats

**Fig. 6:**
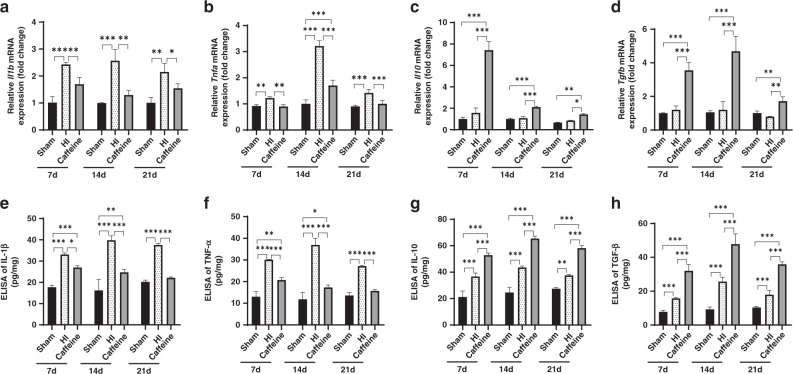
Caffeine regulates cytokine transcription and release in neonatal rats with hypoxic–ischemic WMD. PCR analysis to determine mRNA levels of **a** *Il1b*, **b** *Tnfa*, **c** *Il10*, and **d** *Tgfb*. Levels were normalized against those of *Gapdh* and expressed as fold change. ELISA to determine levels of **e** IL-1β, **f** TNF-α, **g** IL-10, and **h** TGF-β. Data represent the mean ± SEM. Statistical analyses involved two-way ANOVA, followed by Tukey’s test. *P < 0.05, **P < 0.01, ***P < 0.001. Sham group (*n* = 6); HI group (*n* = 6); Caffeine group (*n* = 6). Ca Caffeine.

We then performed RT-PCR to detect the expression of M1- and M2-related cytokines in the animal model on days 7, 14, and 21 after WMD. We found that the expression of M1-related cytokines (*Il1b* and *Tnf1*) in the HI group was higher than that in the sham group (*P* < 0.01, *P* < 0.001, respectively) (Fig. 6a, b); however, there was no difference in terms of M2-related cytokine expression (*Il10* and *Tgfb*) (Fig. 6c, d). Following caffeine treatment, we observed that mRNA levels of M1-related cytokines decreased (*P* < 0.05, *P* < 0.01 or *P* < 0.001, respectively; Fig. 6a, b) and those of M2-related cytokines increased (*P* < 0.05, *P* < 0.01 or *P* < 0.001, respectively) at all time points (Fig. 6c, d). These data indicated that caffeine inhibited the transcription of M1-related cytokines and promoted the transcription of M2-related cytokines.

Additionally, ELISA results revealed significantly higher levels of IL-1β, TNF-α, IL-10, and TGF-β in the brain homogenate of the HI group relative to the sham group (*P* < 0.01, *P* < 0.001, respectively; Fig. 6e–h), with levels of IL-1β and TNF-α significantly decreased and those of IL-10, and TGF-β significantly increased following caffeine administration (*P* < 0.05, *P* < 0.001, respectively) (Fig. 6e–h). These data indicated that caffeine inhibited the release of M1-related proinflammatory factors and promoted the release of M2-related anti-inflammatory factors.

### Caffeine inhibits activation of the NLRP3 inflammasome and regulates microglial polarization by antagonizing A2aR in hypoxic–ischemic WMD

**Fig. 7:**
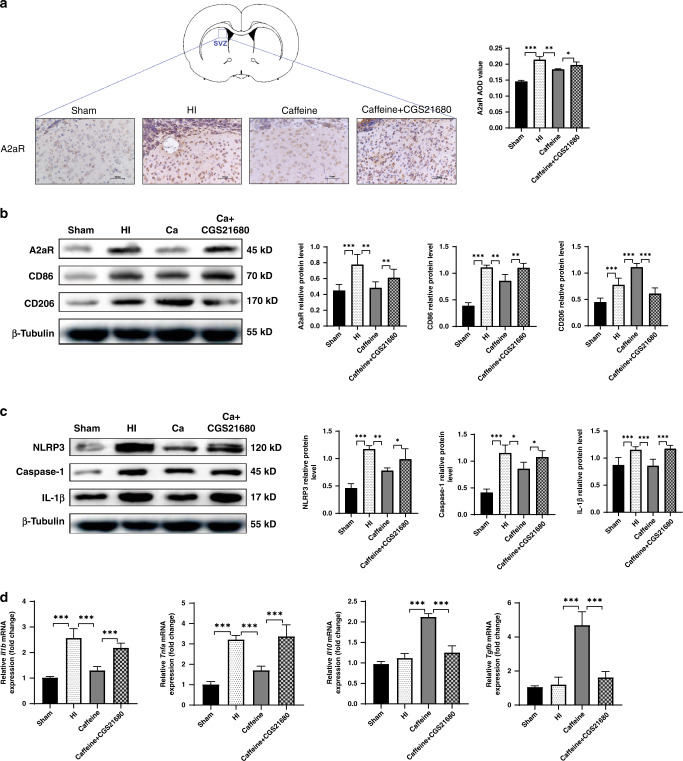
Caffeine inhibits activation of the NLRP3 inflammasome and regulates microglial polarization via A2aR interaction following the establishment of hypoxic–ischemic WMD in neonatal rats. **a** IHC staining for A2aR in the SVZ. Scale bar = 50 μm. Western blot detection of **b** A2aR, CD86, and CD206 and **c** NLRP3, caspase-1, and IL-1β. **d** PCR analysis to determine the mRNA levels of *Il1b*, *Tnfa*, *Il10*, and *Tgfb*. Levels were was normalized against those of *Gapdh* and expressed as fold change. Data represent the mean ± SEM. Statistical analyses involved one-way ANOVA, followed by Tukey’s test. *P < 0.05, **P < 0.01, ***P < 0.001. Sham group (*n* = 6); HI group (*n* = 6); Caffeine group (*n* = 6); Caffeine + CGS21680 group (*n* = 6). Ca Caffeine.

Given the relationship between the caffeine receptor A2aR and neuroinflammation,^33^ we evaluated the role of A2aR in caffeine-mediated inhibition of NLRP3 inflammasome activation and regulation of microglial polarization. IHC and western blot analyses of rats in the caffeine + CGS21680 group showed that at 14 days after WMD establishment, A2aR levels were higher than those in the group treated with caffeine only (*P* < 0.05, Fig. 7a; *P* < 0.01, Fig. 7b). Moreover, western blot results showed that levels of CD86 in the caffeine + CGS21680 group were higher than those in the caffeine group (*P* < 0.01; Fig. 7b), and levels of NLRP3 and NLRP3-related proteins, including caspase-1 and IL-1β, in the caffeine + CGS21680 group were also higher than those in the caffeine group (*P* < 0.05, *P* < 0.001, respectively) (Fig. 7c), whereas levels of CD206 in the caffeine + CGS21680 group were lower than those in the caffeine group at 14 days after WMD establishment (*P* < 0.001; Fig. 7b). These results indicated that caffeine regulated microglial phenotype and inhibited activation of the NLRP3 inflammasome according to A2aR activity.

To confirm these findings, we performed RT-PCR to detect the expression of M1 and M2 cytokines, revealing that levels of M1-related cytokines (*Il1b* and *Tnfa*) in the caffeine + CGS21680 group were elevated relative to those in the caffeine group (*all P* < 0.001), whereas levels of M2-related cytokines (*Il10* and *Tgfb*) in the caffeine + CGS21680 group were lower than those in the caffeine group at 14 days after WMD establishment (all *P* < 0.001) (Fig. 7d). These data suggested that CGS21680 reversed the caffeine-related effects on M1- and M2-related cytokine expression.

## Discussion

We conducted an exploratory study aimed to elucidate the therapeutic effect and mechanism of caffeine on hypoxic–ischemic WMD in premature infants using neonatal rats as an animal model. The results showed that: (1) caffeine had a protective effect against WMD-induced cognitive dysfunction without affecting body weight; (2) caffeine had a protective effect against hypoxic–ischemic WMD and improved myelin sheath-developmental disorders; and (3) caffeine exerted anti-neuro-inflammatory effects, and the mechanism of action included inhibiting NLRP3 inflammasome activation, weakening microglial activation, and promoting microglial polarization toward an anti-inflammatory M2 phenotype.

WMD is a common type of brain damage in premature infants and a primary cause of cerebral palsy and cognitive impairment. It is often caused by cerebral ischemia, hypoxia, and damage related to the inflammatory response.^42,43^ Consistent with previous studies,^37^ we observed spatial learning and memory barriers in our animal model according to the MWM test and subsequently showed that caffeine improved long-term memory in mice, which manifested as a shortening of the escape latency, an increase in the frequency of movement in the target quadrant, an increase in the number of platform crossings, and a decrease in the distance of surrounding activities. Similarly, many experiments in recent years indicated that caffeine has a neuroprotective effect on immature brain damage^27–29,44–46^; however, the specific mechanism remains unclear. Acute hypoxia after unilateral common carotid artery occlusion has been widely used to induce brain WMD.^34^ In the model used in the present study, WMD was consistent with the results of previous studies.^5^ In the normal paraventricular area, bilateral ventricular symmetry is observed; however, in the HI group, we found that both ventricles were enlarged (significantly on the ligated side), and that the left and right ventricles were asymmetric, whereas caffeine treatment improved the degree of enlargement in ventricles. Furthermore, we found that MBP expression was reduced in the HI group relative to that in the sham group; however, caffeine treatment resulted in increased numbers of white-matter cells in the paraventricular area and their orderly arrangement, as well as upregulation of MBP expression.

The cascade of neuroinflammation after hypoxia–ischemia causes the degree of WMD after hypoxia-ischemia to far exceed that of the hypoxic–ischemic brain damage.^47,48^ Numerous clinical studies support the role of inflammation in neonatal hypoxic–ischemic brain injury.^49,50^ Previous studies reported elevated levels of the proinflammatory cytokines IL-6, IL-8, and IL-1β in the cerebrospinal fluid of infants with hypoxic–ischemic encephalopathy, with these elevations related to poor neurological prognosis and possibly closely related to the occurrence of cerebral palsy.^51^ Therefore, reducing neuroinflammatory damage is considered an effective strategy for the treatment of neonatal WMD. Microglia are resident macrophages in the CNS that influence brain development, maintenance of the neural environment, and response to injury and repair.^52^ Under normal conditions, microglia are maintained in a relatively quiescent state. In hypoxic–ischemic brain damage, microglia can be activated through different signaling pathways and polarized to either a proinflammatory or anti-inflammatory state (M1 or M2), which can either aggravate brain damage or promote brain repair, respectively, and play an important role in WMD development and cognitive impairment.^53,54^ Therefore, reducing neuroinflammatory damage and correcting imbalanced microglial polarization are considered effective strategies for the treatment of neonatal WMD. In fact, M1 microglia express markers, such as CD16/32 and CD86, and release proinflammatory mediators, such as TNF-α, iNOS, and IL-1β, that promote neuronal death. By contrast, M2 microglia express markers, such as Arg-1, Ym1, and CD206, and produce anti-inflammatory mediators, including IL-4, IL-10, and TGF-β, that promote tissue repair and support neuronal survival.^55^ M1 microglia secrete cytokines and generate reactive oxygen species, which can directly damage oligodendrocytes, resulting in demyelination. By contrast, M2 microglia secrete nutritional factors that promote the migration and differentiation of oligodendrocyte precursor cells, resulting in re-myelination.^56^ Under normal conditions, there is a balance of M1/M2 microglia, whereas microglial polarization becomes imbalanced during hypoxic–ischemic brain injury, with correction of this imbalance playing a role in inhibiting the release of proinflammatory cytokines and reducing neurotoxicity.^53,56^ The present results support this observation, as the WMD model revealed upregulated M1 and M2 markers following model establishment, whereas caffeine treatment suppressed the M1 phenotype and secretion of associated cytokines (TNF-α and IL-1β) while promoting the M2 phenotype and secretion of anti-inflammatory cytokines (IL-10 and TGF-β).

The NLRP3 inflammasome plays an important role in the innate immune system^57^ and regulates various inflammatory responses in the brain,^58,59^ with numerous studies demonstrating relationships to stroke, cerebral hemorrhage, neurodegenerative diseases, and WMD.^60^ Under the pathological conditions of the CNS, microglia are the main source of inflammasome formation. Previous studies suggest that in many neurological diseases, inhibiting inflammasome activation can regulate microglial polarization.^61–63^ In the present study, the results showed that caffeine treatment decreased NLRP3 levels, as well as the levels of IL-1β and caspase-1. Therefore, we speculated that following hypoxic–ischemic WMD, NLRP3 inflammasome in microglia are activated and proinflammatory cytokines released, which regulate further microglial polarization. However, it remains unclear whether the NLRP3 inflammasome is involved in the transformation of the microglial phenotype after hypoxic–ischemic brain injury.

As a methylxanthine drug, caffeine has been used in neonatal intensive care units to treat neonatal hypoxia for >30 years. Clinical studies show that caffeine has a beneficial effect on the immature brain, with this effect dependent on the age at the start of administration, the dose regularly administered, the neurodevelopmental stage at the time of administration, and the duration of exposure.^64^ Interestingly, recent animal studies showed that caffeine treatment inhibited activation of NLRP3 inflammasomes to reduce neuroinflammation mediated by microglia and improve myelin disorders in mice,^65^ and that inhibition of inflammasome activation by caffeine might be related to A2aR.^66^ In the present study, we showed that caffeine inhibited activation of the NLRP3 inflammasome through A2aR, improved myelination, and reduced cognitive impairment. Consistent with these data, other studies reported the beneficial effects of caffeine on inhibiting microglial activation and reducing neuroinflammation, specifically in neurodegenerative diseases and transient ischemia models.^67,68^ Additionally, Di Martino et al.^44^ used a neonatal hypoxic–ischemic brain-injury model to show that a single dose of caffeine after injury increased MBP and expression of microtubule-associated protein 2 and significantly reduced the number of microglia, apoptotic cells, and the expression of proinflammatory cytokines. In the present study, we found that caffeine reduced microglial polarization from an M1 phenotype and promoted transformation to the M2 phenotype following WMD establishment. These findings suggest that caffeine exerts a positive regulatory effect on inflammasome activation and microglial polarization under conditions of hypoxic–ischemic WMD in neonatal rats. However, the potential mechanisms associated with these protective effects remain to be elucidated.

## Conclusion

In this study, we confirmed that caffeine can inhibit NLRP3 inflammasome activation, promote microglial polarization to the M2 phenotype, and protect against WMD caused by hypoxia–ischemia in neonatal rats. The results suggest caffeine as a promising therapeutic tool against WMD in premature neonates for the protection of their cognitive function. However, further research is needed to determine the specific caffeine-related mechanisms associated with these outcomes, as well as the relationship between the NLRP3 inflammasome and microglial polarization. These findings offer a new scientific basis for the future clinical application of caffeine.

## Supplementary information

Supplementary Material

Supplementary Material
