# Supplementary material for: Caffeine treatment started before injury reduces hypoxic–ischemic white-matter damage in neonatal rats by regulating phenotypic microglia polarization
Source: Pediatr Res. 2022 Feb 26;92(6):1543–54. doi: 10.1038/s41390-021-01924-6 (PMC9771815; doi:10.1038/s41390-021-01924-6)
Supplement: Supplementary file 1 — Supplementary Material [file 41390_2021_1924_MOESM1_ESM.pdf]

A. Grouping diagram

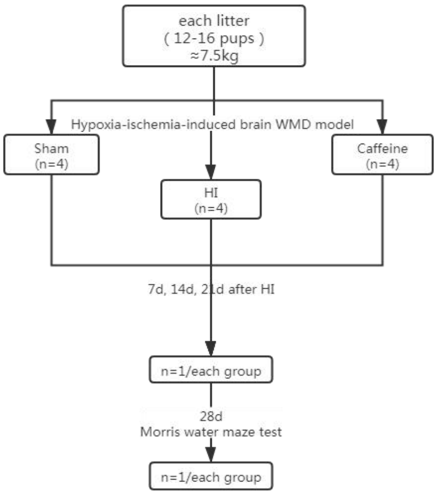

B. Drawing diagram

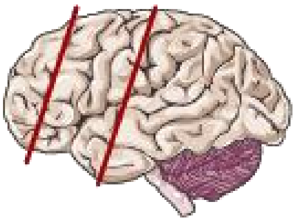

C. The statistical chart of birth weight of each animal in each group

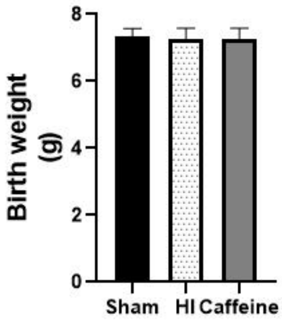

D. The statistical chart of gender of each animal in each group

group * sex * day Crosstabulation

Count

day			sex 0	sex 1	Total
14	group	Caffeine	19	21	40
		HI	20	20	40
		Sham	19	21	40
	Total		58	62	120
21	group	Caffeine	19	21	40
		HI	20	20	40

		Sham	19	21	40
	Total		58	62	120
28	group	Caffeine	3	7	10
		HI	5	5	10
		Sham	5	5	10
	Total		13	17	30
7	group	Caffeine	19	21	40
		HI	21	19	40
		Sham	16	24	40
	Total		56	64	120
Total	group	Caffeine	60	70	130
		HI	66	64	130
		Sham	59	71	130
	Total		185	205	390
