# Supplementary figures and images for: Caffeine treatment started before injury reduces hypoxic–ischemic white-matter damage in neonatal rats by regulating phenotypic microglia polarization
Source: Pediatr Res. 2022 Feb 26;92(6):1543–54. doi: 10.1038/s41390-021-01924-6 (PMC9771815; doi:10.1038/s41390-021-01924-6)

Sham

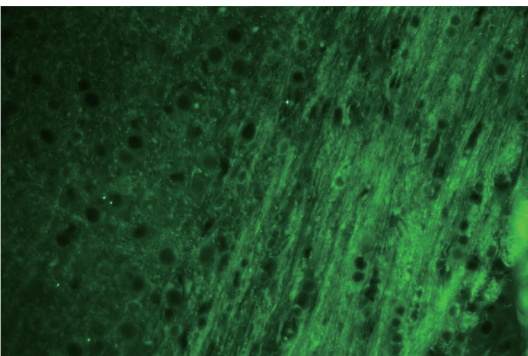

HI

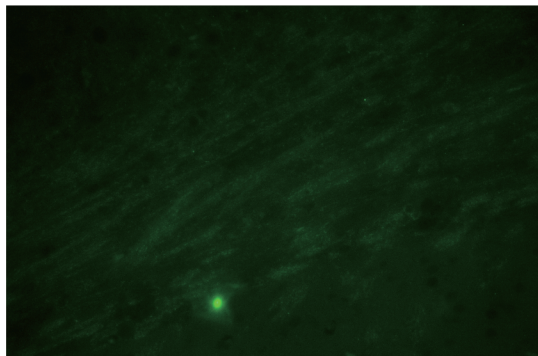

Caffeine (10mg/kg)

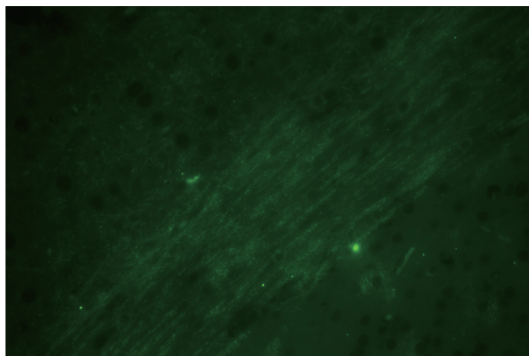

Caffeine (20mg/kg)

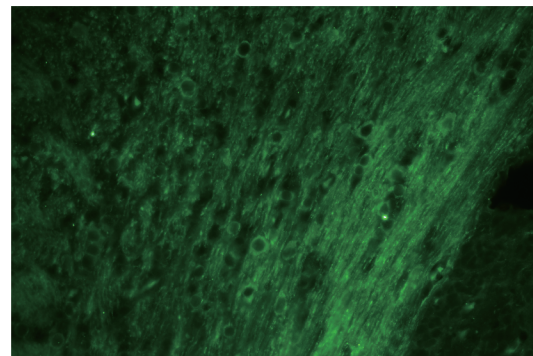

Caffeine (50mg/kg)

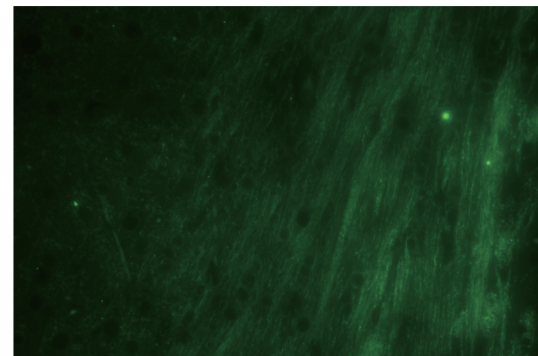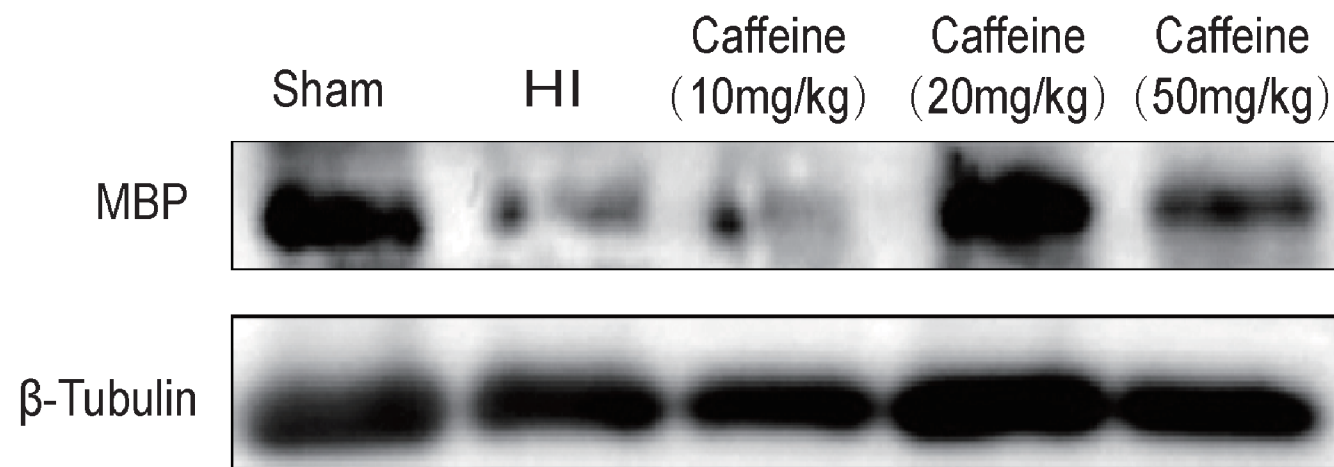

Supplement: Supplementary file 2 — Supplementary Material [file 41390_2021_1924_MOESM2_ESM.pdf]
